# Maryland Hospital for the Insane—Reports for the Year 1850

**Published:** 1851-04

**Authors:** 


*Maryland Hospital for the Insane—Reports for the Year* 1850.—The reports for the last year are altogether favorable. This is an admirable institution, scientifically and *benevolently* managed. It deserves the fostering care of our citizens, and for the honor of the state and the state of humanity, should be enlarged to double its already large capacity.

